# Primary and secondary supportive partnerships among HIV-positive and HIV-negative middle-aged and older gay men

**DOI:** 10.1371/journal.pone.0245863

**Published:** 2021-02-17

**Authors:** Matthew Statz, Deanna Ware, Nicholas Perry, David Huebner, Christopher Cox, Andre Brown, Steven Meanley, Sabina Haberlen, James Egan, Mark Brennan, Linda A. Teplin, Robert Bolan, M. Reuel Friedman, Michael Plankey

**Affiliations:** 1 Georgetown University School of Medicine, Washington, DC, United States of America; 2 Department of Medicine, Division of Infectious Diseases, Georgetown University Medical Center, Washington, DC, United States of America; 3 Warren Alpert Medical School, Brown University, Providence, RI, United States of America; 4 Rhode Island Hospital, Providence, RI, United States of America; 5 Department of Prevention and Community Health, Milken Institute of Public Health, George Washington University, Washington, DC, United States of America; 6 Department of Epidemiology, Bloomberg School of Public Health, Johns Hopkins University, Baltimore, MD, United States of America; 7 Department of Behavioral and Community Health Sciences, Graduate School of Public Health, University of Pittsburgh, Pittsburgh, PA, United States of America; 8 Department of Family and Community Health, University of Pennsylvania School of Nursing, Philadelphia, PA, United States of America; 9 Brookdale Center for Healthy Aging, Hunter College, City University of New York, New York, NY, United States of America; 10 Feinberg School of Medicine, Northwestern University, Chicago, IL, United States of America; 11 Los Angeles LGBT Center, Los Angeles, CA, United States of America; 12 Department of Infectious Diseases and Microbiology, Graduate School of Public Health, University of Pittsburgh, Pittsburgh, PA, United States of America; Instituto Universitário de Lisboa (ISCTE-IUL), CIS-IUL, PORTUGAL

## Abstract

This study describes the primary and secondary partnerships of aging gay men participating in the Understanding Patterns of Healthy Aging Among Men Who Have Sex with Men substudy of the Multicenter AIDS Cohort Study and examines differences in the prevalence of these relationship structures by HIV status while adjusting for age, education, and race/ethnicity. Relationships were compared within the following structural categories: “only a primary partnership”, “only a secondary partnership”, “both a primary and secondary relationship”, or “neither a primary nor secondary relationship”. There were 1,054 participants (51.9% HIV negative/48.1% HIV positive) included in the study. Participants had a median age of 62.0 years (interquartile range: 56.0–67.0) and most reported being non-Hispanic white (74.6%) and college educated (88.0%). Of the 1,004 participants with available partnership status data, 384 (38.2%) reported no primary or secondary partnerships, 108 (10.8%) reported secondary-only partnership, 385 (38.3%) reported primary-only partnership, and 127 (12.6%) reported both primary and secondary partnerships. Of participants who reported primary partnerships only, the prevalence rates (PRs) were lower among those 62 years and older, HIV positive, black non-Hispanic and Hispanics. Of participants who reported only having a secondary partnership, the PRs were higher among those 62 years and older and HIV positive. Of participants who did not report having either a primary or secondary partnership, the PRs were higher among those 62 years and older, HIV positive, and black non-Hispanic compared with their respective referent groups. There was no significant difference in PRs of having both primary and secondary partnerships by age category, HIV status, race/ethnicity, and education. This study aimed to fill a knowledge gap in the literature regarding both primary and secondary supportive partnerships among aging HIV-positive and HIV-negative gay men.

## Introduction

Despite the number of new HIV infections within the US stabilizing over the last few years, [1] improvements in antiretroviral therapy and patient longevity have resulted in a significant increase in the number of Americans older than the age of 50 years living with HIV. In a 2019 report, the Centers for Disease Control and Prevention noted such individuals comprised, at the end of 2016, nearly half of all diagnosed cases in the country [2,3]. Gay men remain disproportionately affected by this disease, with 66% of new diagnoses resulting from male-male sexual transmission, and approximately 60% of HIV-positive men older than age 50 identifying as gay [3]. As these men age, it is critical for the field to improve our understanding of the psychosocial factors that influence their capacity to age well.

For example the importance of psycho-social support and close social ties have been well established in the management of chronic illness, including HIV [4–8]; loneliness has been shown to be linked with an increase in Major Depression and poorer adaptation to illness [9–11]. Previous research has examined types of close relationships independently for aging, sexual minority, and HIV-infected populations [11–14]. However, there is a dearth of studies that jointly investigate these populations. Exploring these factors together may uncover differences in types of close relationships relationship unique to older sexual minority men that impact health, such as the management of chronic illness such as depression and loneliness [9–11] and provide insight into how to better serve the health needs of these men.

It is well established that gay men predominantly seek and engage in committed, long-term relationships with one primary partner similar to the socially accepted heterosexual model [15–17]. However, there is also evidence in the literature demonstrating a portion of gay men are engaged in relationships non-adherent to “traditional” two-person monogamous arrangements [18–20]. Support is also derived from other types of relationships in these men’s lives apart from a primary romantic partner. For example, previous studies have established that family remains an important source of supportive interpersonal relationships regardless of sexual orientation [21,22]. Lesbians and gay men have historically deviated from the heteronormative definition of family as strictly of a biological nature by redefining the term to include individuals who fill that role in the absence or as an extension of biological family [i.e., chosen families] [12,23,24]. Early work by Weston (1991) helped to establish the idea of chosen families as an integral aspect of the support networks of gay men [25]. Due to a lack of social and familial acceptance, possibly exacerbated by HIV-related ostracization, support roles traditionally considered the responsibility of one’s biological family are often filled by partners, friends, and LGBT community organizations [11,12,22,23,26,27]. Indeed, various forms of support have been well established in the literature such as financial, instrumentative, or social support [4,28]. There are numerous typologies that break down forms of support into different hierarchies. Instrumentative support is generally understood as providing assistance, such as helping someone get their groceries, driving them to appointments, etc. whereas social support is understood to include emotional connectedness, “…having someone to talk to about problems, having a sense of belonging or companionship” [4].

Previous studies have explored the socialization and support of older gay men. Grossman and colleagues’ study empirically challenged two pervasive myths: that older gay men are socially isolated and that they remain estranged from their biological families [14]. Shippy et al. supported Grossman and colleagues’ aforementioned findings that older gay men tend to maintain ties to their biological family, but noted that the degree to which biological family was relied on varied [13]. Despite most participants responding they had a parent or sibling still living with whom they were in regular contact, the majority of the men in the study by Shippy and colleagues stated they would first turn to a partner or close friend for most of their social and emotional needs. Aiming to describe the social networks of these older gay men with a focus on caregiving activities, Shippy et al. characterized the elements of the social networks used by middle-aged and aging gay men to address their various support needs (social, emotional, financial, and instrumental). They found that most men were single and lived alone, with the majority reporting at least one close friend within a broad social circle [8,13].

The long-term survival of HIV-positive gay men and some notable societal and legal changes prompt a contemporary recharacterization of the typologies of partnerships among middle-aged and aging gay men living with and without HIV. As Barker et al. noted in their 2006 review: “Very little is known about the pattern over the life course of temporary or permanent partnerships among gay men or lesbians, and there is even a poor understanding of what the term *partner* encompasses for them” [23]. In this current study, we describe the patterns and prevalence of primary and secondary partnerships and whether these relationships differ by HIV status and race/ethnicity using data collected from the Healthy Aging substudy of the Multicenter AIDS Cohort Study (MACS), a longstanding observational cohort of HIV-positive and HIV-negative gay/bisexual men in the US.

## Materials and methods

### Study population

The MACS is an observational investigation that began in 1984 to assess the pathophysiologic factors associated with the natural progression of HIV/AIDS. The study has enrolled 7,352 HIV-positive and -negative men over four distinct waves of enrollment (4,954 in 1984–1985; 668 in 1987–1991; 1,350 in 2001–2003; and 380 in 2010-present) from Baltimore, MD/Washington, DC; Chicago, IL; Los Angeles, CA; and Pittsburgh, PA. Study visits are semiannual and consist of a medical history interview, physical examination, completion of mental and behavioral health questionnaires, and the collection of biospecimens that are stored in a central repository. The MACS study design has been extensively described elsewhere [29,30]. The study instruments can be found at http://www.aidscohortstudy.org. The current analysis uses data from the Healthy Aging substudy, conducted within the MACS core protocol. Eligibility was determined by the completion of at least two consecutive MACS visits immediately preceding April 2016, being at least 40 years of age, and at least one incidence of sexual intercourse with another male since enrolling in the MACS [31]. The analytic sample for this study includes 1,054 men who self-identified as gay and completed a survey during either visit 67 (April 2017-September 2017) or 68 (October 2017-April 2018). If both visits were completed, data from visit 67 was used for analysis. The institutional review boards at John Hopkins University, Northwestern University, University of California Los Angeles, and University of Pittsburgh approved the protocol, and written informed consent was obtained from all study participants.

### Outcome measures

Study participants were asked to answer questions about any primary and secondary partnerships who provide them with emotional or functional support.

#### Primary partnerships

A *primary partner* was defined as someone who the participant is committed to above anyone else—typically in a romantic manner—with whom they might or might not be having sex. The primary partner’s gender (male, female, or transgender), and sexual orientation (gay, lesbian, bisexual, straight/heterosexual, other, prefer not to say, don’t know, or unsure) and the duration of the partnership (in years) were reported. The men were also asked to define the legal status of their primary partnership as one of the following: (1) legally married; (2) registered domestic partnership; (3) unmarried with legal protections (i.e., will, contract, mortgage, or insurance policy); or (4) unmarried without legal protections. “Prefer not to say” and “don’t know/unsure” responses for sexual orientation were later combined into the “other” category.

#### Secondary partnerships

A *secondary partner* was defined as someone with whom the participant might share a bond possibly as intimate or supportive as found in their primary relationship, but that might be strictly platonic or familial. A secondary partnership was described as potentially including the sharing of financial resources, cohabitation, shared histories, or caregiving activities. The secondary partner’s gender (male, female, or transgender), and sexual orientation (gay, lesbian, bisexual, straight/heterosexual, other, prefer not to say, don’t know, or unsure) and the duration of the partnership (in years) were reported. Respondents were further asked to select one or more descriptors that characterized the nature of that individual (secondary partner type): (1) biological family; (2) chosen family; (3) polyamorous/additional romantic partner; (4) close friend; (5) former romantic partner; and (6) current or former sexual partner. An additional category was derived for any participant who reported that their secondary partner was a close friend and a former romantic partner (close friend/former partner).

#### Partnership status

Partnership status was derived from reported primary and secondary partnerships and categorized as follows: (1) no primary or secondary partners; (2) secondary partner and no primary partner; (3) primary partner and no secondary partner; and (4) both primary and secondary partners.

### Covariates

#### Demographic characteristics

Age was calculated from self-reported date of birth and survey completion date and reported as a continuous value in years. In the model, age was categorized into a dichotomous variable (≥ median value: 1; < median value: 0). Race/ethnicity was categorized as: (1) white non-Hispanic; (2) black non-Hispanic; (3) Hispanic; and (4) other (which included American Indian, Asian/Pacific Islander and other racial/ethnic groups not specified). Education was categorized as: (1) less than high school; (2) high school diploma; (3) some or completed college; and (4) graduate school or higher. Sexual orientation was self-reported by the participant as one of the following: (1) gay; (2) lesbian; (3) bisexual; (4) straight/heterosexual; (5) other; (6) prefer not to say; or (7) don’t know/unsure. “Prefer not to say” and “don’t know/unsure” responses were later combined into the “other’” category. Participants selecting any sexual orientation other than gay were removed from the analysis.

#### HIV status

HIV status (HIV positive/HIV negative) was assessed using enzyme-linked immunosorbent assay with confirmatory Western blot. HIV-positive participants included those diagnosed as such at baseline and anyone who seroconverted during study observation.

### Statistical analysis

Descriptive statistics were generated overall and by partnership status for each of the participant’s characteristics (HIV status, age, race/ethnicity, education, and sexual orientation) using frequencies/percentages and medians/interquartile ranges (IQR) as appropriate. In addition, characteristics (partner gender, sexual orientation, and duration of partnership) of primary partnerships by legal status and secondary partnerships by partner type were reported. Participants were able to select 1 or more descriptors for their secondary partner. If multiple descriptors were selected for a single secondary partner, it was set to missing for analysis purposes (with the exception of the additional category of “close friend/former partner”). Duration of relationship by HIV status of the participant and category was reported and compared statistically using nonparametric test of medians.

We modeled partnership status as a multinomial outcome by constructing the log-likelihood and specifying a general distribution using the SAS procedure PROC NLMIXED. The adjusted multinomial model included age category (referent: < median value), HIV status (referent: HIV negative), race/ethnicity (referent: white non-Hispanic), and education status (referent: graduate school) as covariates. Prevalence rates (and their 95% confidence intervals) were computed from the estimated parameters of the multinomial model. Differences in prevalence rates within covariates were tested using Wald tests. Statistical significance was set at <0.05. All statistical analyses were performed using Statistical Analysis Software (SAS) version 9.4 (SAS Institute Inc., Cary, North Carolina, USA).

## Results

### Descriptive statistics

There were 1,054 participants (51.9% HIV negative/48.1% HIV positive) included in the analysis. The median age of participants was 62.0 years (IQR: 56.0–67.0) (Table 1). Most participants reported being white non-Hispanic (74.6%) and having completed at least some college (88.0%). Participants reporting only a primary partnership were mostly HIV negative (62.1%), were primarily white non-Hispanic (84.4%), had at least some college education (92.2%), and had a median age of 61.0 years (IQR: 57.0–66.0). Among participants reporting only a secondary partnership, approximately half were HIV positive (53.7%), primarily white non-Hispanic (76.9%), had at least some college education (85.1%), and had a median age of 64.5 years (IQR: 57.0–69.0). Of participants reporting no partnerships, approximately were HIV positive (51.8%), primarily white non-Hispanic (70.6%), had at least some college education (87.5%), and had a median age of 62.0 years (IQR: 56.0–68.0). Of participants reporting both primary and secondary partnerships, approximately half were HIV positive (54.3%), mostly white non-Hispanic (70.1%), had at least some college education (86.6%), and had a median age of 61.0 years (IQR: 54.0–67.0). Further details on covariate distribution by partnership status are reported in Table 1.

**Table 1:** Demographic characteristics by partnership status.

	Partnership status					
	Primary only (n = 385)	Secondary only (n = 108)	No primary or secondary (n = 384)	Both primary and secondary (n = 127)	Missing partnership status (n = 50)	Total (N = 1054)
**HIV status of participant, n (%)**						
Negative	239 (62.1%)	50 (46.3%)	185 (48.2%)	58 (45.7%)	15 (30.0%)	547 (51.9%)
Positive	146 (37.9%)	58 (53.7%)	199 (51.8%)	69 (54.3%)	35 (70.0%)	507 (48.1%)
**Age (years), median (IQR)**	61.0 (57.0, 66.0)	64.5 (57.0, 69.0)	62.0 (56.0, 68.0)	61.0 (54.0, 67.0)	56.5 (51.0, 65.0)	62.0 (56.0, 67.0)
**Race/ethnicity, n (%)**						
White non-Hispanic	325 (84.4%)	83 (76.9%)	271 (70.6%)	89 (70.1%)	18 (36.0%)	786 (74.6%)
Black non-Hispanic	30 (7.8%)	13 (12.0%)	73 (19.0%)	19 (15.0%)	20 (40.0%)	155 (14.7%)
Hispanic	24 (6.2%)	10 (9.3%)	33 (8.6%)	16 (12.6%)	10 (20.0%)	93 (8.8%)
Other	6 (1.6%)	2 (1.9%)	7 (1.8%)	3 (2.4%)	2 (4.0%)	20 (1.9%)
**Education, n (%)**						
Less than high school	3 (0.8%)	1 (0.9%)	11 (2.9%)	1 (0.8%)	5 (10.0%)	21 (2.0%)
High school diploma	27 (7.0%)	15 (13.9%)	37 (9.6%)	16 (12.6%)	10 (20.0%)	105 (10.0%)
Some or completed college	198 (51.4%)	52 (48.1%)	197 (51.3%)	70 (55.1%)	27 (54.0%)	544 (51.6%)
Graduate school or higher	157 (40.8%)	40 (37.0%)	139 (36.2%)	40 (31.5%)	8 (16.0%)	384 (36.4%)

IQR: Interquartile range.

#### Legal status of primary partnerships

A total of 512 participants (48.6%) reported having a primary partner. Among these primary partnerships, 232 (45.3%) were legally married, 23 (4.5%) were registered domestic partners, 111 (21.7%) were unmarried with some legal protections, and 140 (27.3%) were unmarried with no legal protections (Table 2). There were 6 cases (1.2%) missing legal status. Approximately half of the participants who reported primary partners were HIV negative (58.0%). The primary partners were predominantly male (98.0%) and identified as gay (94.9%). Distribution of the primary partners’ gender and sexual orientation was similar across legal status. The overall median duration of primary partnerships was 18.0 years (IQR: 8.7–27.5). Participants with legally married and registered domestic partners had the longest relationship duration at 23.0 years (IQR: 15.5–32.0) and 24.0 (IQR: 16.0–30.0), respectively. Participants who were unmarried with legal protections and unmarried with no legal protections had relationship durations of 17.0 (IQR: 10.0–26.0) and 8.4 years (IQR: 2.3–17.0), respectively. These durations were statistically different by legal status(p<0.0001). Further details on characteristics by legal status are reported in Table 2.

**Table 2:** Characterization of primary partnerships (n = 512) by legal status.

	No primary partner (n = 492)	Legally married (n = 232)	Registered domestic partners (n = 23)	Unmarried, with legal protections (n = 111)	Unmarried, with no legal protections (n = 140)	Missing legal status (n = 6)	Total (N = 512)
**HIV status of participant, n (%)**							
Negative	235 (47.8%)	144 (62.1%)	13 (56.5%)	65 (58.6%)	75 (53.6%)	0 (0.0%)	297 (58.0%)
Positive	257 (52.2%)	88 (37.9%)	10 (43.5%)	46 (41.4%)	65 (46.4%)	6 (100.0%)	215 (42.0%)
**Gender of primary partner, n (%)**							
Male	-	228 (98.3%)	21 (91.3%)	110 (99.1%)	138 (98.6%)	5 (83.3%)	502 (98.0%)
Female	-	3 (1.3%)	0 (0.0%)	0 (0.0%)	1 (0.7%)	0 (0.0%)	4 (0.8%)
Missing	-	1 (0.4%)	2 (8.7%)	1 (0.9%)	1 (0.7%)	1 (16.7%)	6 (1.2%)
**Sexual orientation of primary partner, n (%)**							
Gay	-	226 (97.4%)	22 (95.7%)	105 (94.6%)	128 (91.4%)	5 (83.3%)	486 (94.9%)
Lesbian	-	0 (0.0%)	0 (0.0%)	1 (0.9%)	0 (0.0%)	0 (0.0%)	1 (0.2%)
Bisexual	-	2 (0.9%)	1 (4.3%)	3 (2.7%)	7 (5.0%)	0 (0.0%)	13 (2.5%)
Straight/Heterosexual	-	2 (0.9%)	0 (0.0%)	0 (0.0%)	4 (2.9%)	0 (0.0%)	6 (1.2%)
Missing	-	2 (0.9%)	0 (0.0%)	1 (0.9%)	1 (0.7%)	1 (16.7%)	5 (1.0%)
**Duration of relationship (years), median (IQR)**		23.0 (15.5, 32.0]	24.0 (16.0, 30.0)	17.0 (10.0, 26.0)	8.4 (2.3, 17.0)	11.6 (2.5, 14.0)	18.0 (8.7, 27.5)
**Duration of relationship (years) by HIV status, median (IQR)**							
HIV positive	-	20.5 (10.1–29.5)	20.9 (16.0–27.0)	14.2 (8.3–24.0)	7.0 (2.0–18.0)	-	15.0 (6.0–24.1)
HIV negative	-	25.2 (17.1–33.2)	25.0 (24.0–31.0)	18.0 (12.0–31.0)	10.0 (3.0–16.0)	11.6 (2.5–14.0)	6.0 (15.0–24.1)

IQR: Interquartile range.

#### Types of secondary partnerships

A total of 235 participants (22.3%) reported having a secondary partner. The reported secondary partner types were close friends (34.0%), former romantic partners (16.6%), biological family (8.5%), chosen family (5.5%), current/former sexual partner (6.4%), close friend who was also a former romantic partner (2.1%), and additional romantic partner (3.0%). There were 56 cases (23.8%) missing secondary partner types. Most of the secondary partners were male (81.7%) and identified as gay (69.8%). The distribution of these characteristics by secondary partner type was similar, with the exception of the sexual orientation of biological family, where 85.0% were reported to be straight/heterosexual. The median duration of partnership was 24.0 years (IQR: 12.0–35.0). Biological family had the longest partnership duration at 53.5 years (IQR: 49.0–63.0). The median duration of chosen family, additional partner, close friend, close friend/former partner, former partner and current/former sexual partner were 24.0 years (IQR:11.0–35.0), 15.0 (IQR: 2.0–20.0), 23.5 years (IQR: 10.5–33.6), 23.0 years, (IQR: 17.0–30.0), 26.3 years, (IQR: 17.0–35.0), and 15.0 years, (IQR: 3.0–30.0), respectively. These durations were statistically different (p<0.0001). Further details on the characteristics by partner type are reported in Table 3.

**Table 3:** Characterizations of secondary partnerships by partnership type (n = 235).

	No secondary partner (n = 769)	Biological family (n = 20)	Chosen family (n = 13)	Additional partner (n = 7)	Close friend (n = 80)	Close friend/former partner (n = 5)	Former partner (n = 39)	Current/ former sexual partner (n = 15)	Missing partner type (n = 56)	Total (N = 235)
**HIV status of participant, n (%)**										
Negative	424 (55.1%)	5 (25.0%)	8 (61.5%)	6 (85.7%)	31 (38.8%)	1 (20.0%)	20 (51.3%)	8 (53.3%)	29 (51.8%)	108 (46.0%)
Positive	345 (44.9%)	15 (75.0%)	5 (38.5%)	1 (14.3%)	49 (61.3%)	4 (80.0%)	19 (48.7%)	7 (46.7%)	27 (48.2%)	127 (54.0%)
**Gender of secondary partner, n (%)**										
Male	-	8 (40.0%)	9 (69.2%)	7 (100.0%)	60 (75.0%)	4 (80.0%)	38 (97.4%)	15 (100.0%)	51 (91.1%)	192 (81.7%)
Female	-	12 (60.0%)	4 (30.8%)	0 (0.0%)	19 (23.8%)	1 (20.0%)	0 (0.0%)	0 (0.0%)	3 (5.4%)	39 (16.6%)
Transgender	-	0 (0.0%)	0 (0.0%)	0 (0.0%)	0 (0.0%)	0 (0.0%)	0 (0.0%)	0 (0.0%)	1 (1.8%)	1 (0.4%)
Missing	-	0 (0.0%)	0 (0.0%)	0 (0.0%)	1 (1.3%)	0 (0.0%)	1 (2.6%)	0 (0.0%)	1 (1.8%)	3 (1.3%)
**Sexual orientation of secondary partner, n (%)**										
Gay	-	1 (5.0%)	7 (53.8%)	6 (85.7%)	54 (67.5%)	4 (80.0%)	37 (94.9%)	13 (86.7%)	42 (75.0%)	164 (69.8%)
Lesbian	-	1 (5.0%)	0 (0.0%)	0 (0.0%)	2 (2.5%)	0 (0.0%)	0 (0.0%)	0 (0.0%)	0 (0.0%)	3 (1.3%)
Bisexual	-	1 (5.0%)	0 (0.0%)	1 (14.3%)	3 (3.8%)	0 (0.0%)	1 (2.6%)	1 (6.7%)	5 (8.9%)	12 (5.1%)
Straight/Heterosexual	-	17 (85.0%)	6 (46.2%)	0 (0.0%)	20 (25.0%)	1 (20.0%)	1 (2.6%)	0 (0.0%)	5 (8.9%)	50 (21.3%)
Other*	-	0 (0.0%)	0 (0.0%)	0 (0.0%)	1 (1.3%)	0 (0.0%)	0 (0.0%)	0 (0.0%)	3 (5.4%)	4 (1.7%)
Missing	-	0 (0.0%)	0 (0.0%)	0 (0.0%)	0 (0.0%)	0 (0.0%)	0 (0.0%)	1 (6.7%)	1 (1.8%)	2 (0.9%)
**Duration of relationship (years), median (IQR)**		53.5 (49.0–63.0)	24.0 (11.0–35.0)	15.0 (2.0–20.0)	23.5 (10.5–33.6)	23.0 (17.0–30.0)	26.3 (17.0–35.0)	15.0 (3.0–30.0)	20.0 (10.0–35.0)	24.0 (12.0–35.0)
**Duration of relationship (years), median (IQR)**										
HIV positive		54.0 (50.0–63.0)	11.0 (8.0–20.0)	21.0 (21.0–21.0)	24.0 (11.0–30.0)	20.0 (13.5–29.0)	24.0 (17.0–35.0)	6.0 (3.2–20.0)	20.0 (9.0–35.0)	25.0 (12.0–36.0)
HIV negative		50.0 (42.0–63.0)	28.5 (19.5–42.5)	9.0 (2.0–16.0)	20.0 (6.0–37.0)	30.0 (30.0–30.0)	33.0 (16.8–36.5)	27.5 (2.5–31.5)	20.1 (10.0–35.0)	23.1 (11.0–35.0)

IQR: Interquartile range.

*Includes other, N/A, and prefer not to say responses.

#### Overall partnership prevalence rates

There were 1,004 participants with available partnership status data. Of these participants, 384 (38.2%) reported no primary or secondary partnerships, 108 (10.8%) reported secondary-only partnership, 385 (38.3%) reported primary-only partnership, and 127 (12.6%) reported both primary and secondary partnerships. The distribution of age, race/ethnicity, and HIV status differed significantly by partnership status (Table 1).

#### Partnership multinomial model

*Participants who did not have primary or secondary partnerships*. Of participants who did not report having either a primary or secondary partnership, the prevalence rates (PRs) were higher at 35.3% among those 62 years and older (95% CI: 29.8%-40.9%; p = 0.0095), 34.9% among those who were HIV positive (95% CI: 27.3%-42.6%; p = 0.0130), and 47.4% among black non-Hispanics (95% CI: 35.6%-59.2%; p<0.001) compared with their respective referent groups (PR: 27.3%; 95% CI: 20.7%-33.9%) (Table 4).

**Table 4:** Adjusted prevalence rates (95% confidence intervals) of the number of partnerships.

	Adjusted prevalence rates (95% confidence intervals)			
	Primary partnership only	Secondary partnership only	No primary or secondary partnerships	Both primary and secondary partnerships
**Age category**				
≥ 62 years old	44.3% (38.4% - 50.2%)^a^	10.8% (7.2% - 14.5%)^a^	35.3% (29.8% - 40.9%)^a^	9.6% (6.3% - 12.9%)
< 62 years old (referent)	58.9% (51.2% - 66.6%)	5.2% (2.4% - 7.9%)	27.3% (20.7% - 33.9%)	8.6% (4.7% - 12.5%)
**HIV status**				
Positive	45.2% (36.9% - 53.5%)^a^	8.1% (4.0% - 12.2%)^a^	34.9% (27.3% - 42.6%)^a^	11.8% (6.6% - 16.9%)
Negative (referent)	58.9% (51.2% - 66.6%)	5.2% (2.4% - 7.9%)	27.3% (20.7% - 33.9%)	8.6% (4.7% - 12.5%)
**Race/ethnicity**				
Black non-Hispanic	36.1% (24.4% - 47.9%)^a^	5.4% (1.4% - 9.5%)	47.4% (35.6% - 59.2%)^a^	11.0% (4.3% - 17.7%)
Hispanic	44.6% (30.5% - 58.6%)^a^	7.2% (1.6% - 12.9%)	33.6% (21.3% - 46.0%)	14.6% (5.6% - 23.5%)
Other	46.6% (20.8% - 72.5%)	6.7% (0.0% - 16.5%)	33.9% (11.1% - 56.6%)	12.8% (0.0% - 27.7%)
White, non-Hispanic (referent)	58.9% (51.2% - 66.6%)	5.2% (2.4% - 7.9%)	27.3% (20.7% - 33.9%)	8.6% (4.7% - 12.5%)
**Education**				
Less than high school	39.2% (7.9% - 70.4%)	5.2% (2.4% - 7.9%)	52.1% (22.6% - 81.7%)	4.6% (0.0% - 13.9%)
High school diploma	47.3% (34.5% - 60.0%)	4.1% (0.0% - 12.3%)	29.5% (19.2% - 39.9%)	13.2% (5.7% - 20.7%)
Some or completed college	55.6% (48.7% - 62.5%)	10.0% (3.7% - 16.3%)	28.3% (22.4% - 34.2%)	10.5% (6.6% - 14.5%)
Graduate school (referent)	58.9% (51.2% - 66.6%)	5.2% (2.4% - 7.9%)	27.3% (20.7% - 33.9%)	8.6% (4.7% - 12.5%)

^a^p<0.05 denotes statistically significant difference from covariate referent group.

*Participants who had secondary partnerships only*. Of participants who reported only having a secondary partnership, the PRs were higher at 10.8% among those 62 years and older (95% CI: 7.2% - 14.5%; p = 0.0003) and 8.1% among those who were HIV positive (95% CI: 4.0%-12.2%; p = 0.0464) compared with their respective referent groups (PR: 5.2%; 95% CI: 2.4%-7.9%) (Table 4).

*Participants who had primary partnerships only*. Of participants who reported primary partnerships only, the PRs were lower at 44.3% among those 62 years and older (95% CI: 38.4%-50.2%; p<0.0001), 45.2% among those who were HIV positive (95% CI: 36.9%-53.5%; p<0.0001), 36.1% among black non-Hispanics (95% CI: 24.4%-47.9%; p<0.001), and 44.6% among Hispanics (95% CI: 30.5%-58.6%; p = 0.0306) compared with their respective referent groups (PR: 58.9%; 95% CI: 51.2%-66.6%) (Table 4).

*Participants who had both primary and secondary partnerships*. There was no significant difference in PRs of having both primary and secondary partnerships by age category, HIV status, race/ethnicity, and education (Table 4).

## Discussion

This study, to our knowledge, is the first study to examine differences in typologies of primary and secondary partnerships among gay based on HIV status. Although nearly half of the men in this study reported having a primary partner, less than a quarter of participants reported having a secondary supportive partner. Few men reported having both a primary and secondary partner or a secondary partner only. Of the men in a primary relationship, most were HIV negative. Older gay men tended not to be married to their primary partners but often still reported some degree of legal protection; only a quarter of the men stated that their relationship lacked legal formality entirely. Most of the participants’ primary partners identified as gay men. For both primary and secondary partnerships, the relationships tended to be long-standing. Contrary to the findings of previous studies [8,13], the men listed their biological family as their secondary source of support more frequently than someone of their chosen family. Nearly one in five men who provided information about their secondary partner listed a former romantic partner as their secondary source of support.

Perhaps most concerning was the prevalence of older gay men who reported having neither a primary nor secondary supportive relationship. Race, age, and HIV status were all independently associated with this category. Black non-Hispanic men reported lacking both primary and secondary supportive partners at significantly greater rates than men of other races and ethnicities, as were men over the age of 61, and men who were HIV-positive. Our findings are consistent with Emlet’s 2006 study exploring the social networks and social isolation of HIV-positive men, which found that men of color scored significantly lower both in terms of number of friendships and on questions of instrumental support, as well as the number of friends who met the threshold for “functional support” established in the literature, which is based on both the frequency and nature of contact [32–34].

The findings of previous studies offer some potential explanations for this lack of supportive relationships among black middle-aged and older gay men. One explanation is the limited number of available partners for black gay men. High rates of incarceration and premature death among black men combined with greater rates of intraracial dating among black people has led to a smaller pool of available black men with whom to form romantic and supportive relationships [35–38]. These sociostructural factors can create barriers to the formation and maintenance of supportive relationships and may help to explain this study’s findings regarding middle-aged and older black men lacking supportive relationships [36].

Intersecting social stigmas attached to the racial and sexual identities of black middle-aged and older gay men may also help to explain their lower prevalence of supportive relationships found in this study. Black gay men face pressure to conform to potentially conflicting roles between the expectations of their family and black community in which heteronormativity is expected [23,36,37,39] and the expectations of the LGBT community, which holds its own array of racially specific behavioral stigmas/expectations “such as the ‘aggressive black top’ and the ‘submissive Asian bottom’” [36]. Decades ago, Icard suggested a multivariate framework for assessing the psychosocial well-being of gay black men, arguing that due to cultural norms, many black men have had to prioritize or even compartmentalize their identities as black men and gay men in order to reduce the possibility of ostracization from either community [39]. Recent studies have explored the pressures felt by black gay and bisexual men in regard to the intersectionality of those three characteristics in defining their identity and the difficulty some have had in separating those aspects of themselves in order to maintain relationships in both the LGBT and black communities [40]. Black men have been shown to rely more heavily on biological family for support than other racial/ethnic groups and face increased familial rejection should they come out [32,39,41]. The relative infrequency of supportive relationships amongst older black gay men in our study might be indicative of the difficulty these men have had in navigating these disparate expectations.

HIV status was a statistically significant factor across three of the categorical groups, with HIV-positive men less likely to have a primary partner, and more likely to report having only a secondary source of support or no partner at all. A negative HIV status meant men were more likely to report having a primary partner, but no secondary partner. interestingly, HIV-positive men were three times as likely to list biological family as a secondary partner (n = 15 vs n = 5) than HIV-negative men.

A number of explanations for the differences in relationships’ structural prevalence based on HIV status is worth discussing here. In their 2006 review, Barker and colleagues [27] noted the significant impact the early AIDS epidemic had on the gay community, writing “It was not uncommon for gay men to speak of losing 20 or more close friends or acquaintances to AIDS within a period of 5 or 10 years.” It is entirely conceivable that these HIV-positive men may not have replaced their primary partners who died, and this loss could consequently have strengthened the importance of the support from secondary partners. Independent of source of support, disease coping, practical coping, and emotional coping skills were salient themes expressed by long-term survivors of HIV when asked “What has helped survivors cope with challenges of living long term with HIV?” [42]. As the majority of the participants in this study were long-term survivors, we postulate that the same phenomena are applicable here.

This study’s aim was to explore the current typology of supportive relationships older gay men reported having in their lives and examining whether the types of relationships varied by demographic characteristics and HIV status. This updating of the literature is necessary in part because of broad social changes, such as the nationalization of gay marriage, that significantly alter the availability of legal protections for their partnerships. Many of the studies that looked at similar populations occurred prior to these changes and used different sampling techniques, but some comparison of findings is still relevant.

Grossman and colleagues [14] found that gay men reported friends to be the most prevalent source of support, followed by partners. In contrast, we found the opposite pattern: 46% of all participants reporting a primary partner and only 39% of the 270 men who listed a secondary source of support characterized them as a close friend. Our results provide nuance to the findings of Grossman and colleagues as well as those of Shippy and colleagues [8,13], which both contradicted the generally assumed social isolation of older gay men. While we cannot comment on the breadth or complexity of these men’s social networks overall as described in both studies, it is clear from the number of men reporting no primary or secondary partner that for many of them that does not include an individual, romantic or otherwise, who they felt met our survey’s definition of a supportive partner. It is important to note that additional questions were asked regarding the respondents’ perceived access to support in general, but those findings fell outside the aims of this study.

The relative infrequency at which chosen family was reported as a secondary supportive relationship differs significantly from what established literature has shown. Studies exploring the concept of chosen family and its importance in the LGBT community showed the primary source of support for older gay men was a partner, if present, followed by friends including those considered “chosen family” [23,43]. Once the distinction between “chosen family” and "close friend” is made, the relative importance of chosen-family members as a secondary source of support, compared with the reliance on biological family, is interesting. Such a categorical distinction was not made in previous studies looking at support, making it impossible to determine whether this reflects a change in cultural norms or not.

There are several limitations to this study. Our questions typifying these bonds as primary and secondary relationships are different than how other studies have assessed sources of support for gay men [13,14,32]. It is possible that our data reflect a different interpretation than previous work, complicating comparisons with the literature. It should be noted here that an attempt to make a direct comparison of our findings with those from observational studies of gay men outside the US was very limited. These studies tended to focus on breadth or impact of such mens’ social networks on their mental health and loneliness [4,10,44], rather than provide descriptive data regarding reporting of primary or secondary relationships. Moreover, the language used in our study—defining secondary partners with great specificity—may explain why our findings are different than those of prior studies. Additionally, the questions included in our questionnaire did not adhere to any one strict metric, as our aim was simply to establish the presence or absence of support, as perceived by the subject, from a primary and/or secondary source for these men. As such, our questions used descriptor phrases that broadly included all of the aforementioned categories (“someone who you are committed to above anyone else”, “someone who shares financial resources to pay living expenses, shares housing, shares personal sacred histories between both of you, or takes cares of you when seriously ill (or you them)”.) Furthermore, several questions that were not addressed in the current survey, such as the age and HIV status of the partners, would have been useful to characterize these men’s relationships/sources of support. Nevertheless, the MACS is one of the largest longstanding, well-characterized cohorts of middle-age and aging gay men and, as such, the quality of the data from which this study was performed is exceedingly reliable. However, it is a convenience sample and our results might not be generalizable to the wider population of gay men. Despite these considerations, this work expands the understanding of where older gay men derive their support and the nature of those relationships. Understanding these typologies will help identify omissions in the support these men are receiving.

## Conclusions

This study provides a number of points of interest from which further study could continue. For instance, while less than 50% of the participants with a primary partner reported being legally married, the rate of marriage was notable and behooves further exploration into comparing these findings with rates prior to the Supreme Court’s 2015 decision legalizing same-sex marriage nationwide. Given the extensive literature establishing the importance of social support in chronic illness management, social isolation, and healthy aging, the rate at which these men, especially those with HIV, reported having neither a primary nor secondary partner is particularly concerning. Further investigation of how these structures impact psychosocial resiliencies, loneliness, and other health outcomes are urgently needed. Understanding how these men maintain psychological wellness as they age is contingent on accurate characterizations of the relationships on which they rely for their social and emotional support. The data presented here could be used in subsequent work to further elucidate how such men distinguish between a primary or secondary partner and other supportive elements of their social networks.
